# Perceptions of Grandmothers’ Favoritism: Consequences for Adult Grandchildren’s Well-Being

**DOI:** 10.1093/geroni/igaf122.1896

**Published:** 2025-12-31

**Authors:** Destiny Ogle, J Jill Suitor, Megan Gilligan, Shawn Bauldry

**Affiliations:** Purdue University, West Lafayette, Indiana, United States; Purdue University, West Lafayette, Indiana, United States; University of Missouri, Columbia, Missouri, United States; Purdue University, West Lafayette, Indiana, United States

## Abstract

The consequences of maternal differential treatment on adult children’s psychological well-being are well-documented. However, no consideration has been given to grandmothers’ differentiation among their adult grandchildren and its consequences for the well-being of members of the youngest generation. Drawing from theories of socioemotional selectivity and gender-role socialization, this study uses mixed-methods data collected from 221 adult grandchildren nested within 81 families from the Within-Family Differences Study-III to investigate the association between adult grandchildren’s perceptions of grandmothers’ favoritism and psychological well-being. Forty-nine (28.5%) of the respondents perceived themselves as the grandchildren to whom their grandmothers were most emotionally close. Multilevel regression analyses suggest that perceiving oneself as the grandchild to whom grandmothers were the most emotionally close was associated with higher depressive symptoms among granddaughters, but not grandsons. Qualitative analyses revealed that these gender differences emerged from variations in the meaning granddaughters and grandsons ascribed to being the favored grandchild. In particular, granddaughters were much more likely than grandsons to explain their role as their grandmothers’ emotional caregiver—a role that has been found to contribute to adult daughters’ distress when their mothers face age-related challenges in later life. These findings contribute to the literature on differential treatment in intergenerational families by demonstrating that perceptions of grandmothers’ emotional closeness are both common and consequential for the psychological well-being of adult granddaughters.

